# The influence of anger on empathy and theory of mind

**DOI:** 10.1371/journal.pone.0255068

**Published:** 2021-07-29

**Authors:** Ronja Weiblen, Noam Mairon, Sören Krach, Macià Buades-Rotger, Mor Nahum, Philipp Kanske, Anat Perry, Ulrike M. Krämer

**Affiliations:** 1 Department of Neurology, University of Lübeck, Lübeck, Germany; 2 Department of Psychiatry and Psychotherapy, Translational Psychiatry Unit (TPU), University of Lübeck, Lübeck, Germany; 3 School of Occupational Therapy, The Hebrew University of Jerusalem, Jerusalem, Israel; 4 Center of Brain, Behavior and Metabolism (CBBM), University of Lübeck, Lübeck, Germany; 5 Clinical Psychology and Behavioral Neuroscience, Faculty of Psychology, Technische Universität Dresden, Dresden, Germany; 6 Max Planck Institute for Human Cognitive and Brain Sciences, Leipzig, Germany; 7 Department of Psychology, The Hebrew University of Jerusalem, Jerusalem, Israel; 8 Department of Psychology, University of Lübeck, Lübeck, Germany; Victoria University of Wellington, NEW ZEALAND

## Abstract

Social cognition allows humans to understand and predict other people’s behavior by inferring or sharing their emotions, intentions and beliefs. Few studies have investigated the impact of one’s own emotional state on understanding others. Here, we tested the effect of being in an angry state on empathy and theory of mind (ToM). In a between-groups design we manipulated anger status with different paradigms in three studies (autobiographical recall (N = 45), negative feedback (N = 49), frustration (N = 46)) and checked how this manipulation affected empathic accuracy and performance in the EmpaToM. All paradigms were successful in inducing mild anger. We did not find the expected effect of anger on empathy or ToM performance but observed small behavioral changes. Together, our results validate the use of three different anger induction paradigms and speak for rather weak behavioral effects of mild state anger on empathy and ToM.

## Introduction

In everyday life, being angry is often associated with irrational decision making, with saying things one regrets later and with being unable or unwilling to understand or share another person’s point of view (“blinded by rage”). However, surprisingly few studies have formally tested the relationship between anger and understanding or sharing others’ mental states. Studies have found a negative correlation between empathy and anger expressions in children [1, 2], as well as impaired empathy in men with a history of legally relevant aggressive behavior [3]. However, it is unclear if a person’s current state of anger affects understanding and sharing mental states of others. In the present study, we addressed this question by experimentally manipulating anger status before testing participants’ social cognition in established empathy and theory of mind paradigms (empathic accuracy [4]; EmpaToM [5]). Both anger and social cognition can be studied as a trait, meaning a relatively stable disposition or ability, or a state, meaning the current situational anger or social evaluations [6]. In this paper, we will focus on the state aspect of both concepts.

Social cognition refers to how we think about our own or others’ traits, understand others’ feelings, consider a person’s intentions or take the perspective of others into account [7]. Because humans cannot directly perceive others’ thoughts or feelings, they use their own experiences, thoughts and feelings to understand the internal experiences of others. This involves two complementary systems [8, 9]: *empathy* and *theory of mind (ToM)*. Empathy describes processes in which people vicariously take on affective states, mental states, facial expressions, and postures of others´, literally sharing their experience [10]. These phenomena are also coined affective empathy or experience sharing. When it comes to sharing others´ negative emotional states, Singer and Klimecki [11] distinguish two forms of empathic responses: empathic distress and compassion. When confronted with negative emotions in another person, empathic distress is the emergence of strong, negative, self-related feelings which lead to withdrawal from the situation, while compassion describes positive feelings like warmth and care which are other-related and motivate to approach and help. ToM describes processes in which people take someone else´s perspective by projecting themselves into the situation of others, inferring their thoughts and feelings from the context and their own supposed experience in this situation [8]. This is also described as cognitive empathy or mentalizing.

Both routes to social cognition require distinguishing between one’s own and the other’s mental state. Interestingly though, only few studies investigated the impact of one’s own mental and especially emotional state on social cognition. For example, in a study involving therapists, pre-session positive affect was negatively correlated with empathy during the session, while pre-session anxiety was positively correlated with ToM performance [12]. Furthermore, induced sadness in comparison to happiness was found to facilitate ToM performance [13].

Anger is an emotional state, which varies in intensity from mild annoyance to intense fury and rage [14]. Recent definitions of anger propose a multidimensional construct with four components: a physiological component including general sympathetic arousal and a change in the level of hormones and neurotransmitters; a cognitive component containing irrational beliefs, automatic thoughts and inflammatory imagery; a phenomenological component including the subjective awareness and labeling of angry feelings and a behavioral component containing facial expressions and verbal and behavioral anger expression strategies [15]. Extensive research has examined what triggers anger [16–23]. Berkowitz [24] summarizes that anger is elicited by situations in which people are injured, deceived or betrayed, by situations in which they are physically or psychologically controlled against their will, and by situations where they are prevented from reaching a goal.

Different mechanisms are conceivable through which anger interferes with understanding others. Regarding empathy, the frustrating event preceding the angry state such as goal blocking or personal offense might lead to a higher focus on one’s own needs and thereby reduce compassion and consideration of the others’ state and needs. In line with this, a recent study found that negative mood decreased the neural correlate of empathy for pain (mu suppression) in comparison to neutral or positive mood [25]. Regarding ToM, increased arousal, as common in an angry state, might generally lower cognitive capacities to take the other’s perspective and infer the other’s mental state. In agreement with this account, a series of studies showed reduced perspective taking in angry participants, which was moderated by the magnitude of induced arousal [26]. This is somewhat contradictory to the previously mentioned finding that state anxiety, another emotion characterized by arousal, positively correlated with ToM performance [12]. However, as Litvak and colleagues [27] pointed out in an overview article of the impact of anger on decision-making, the effects of anger on cognition seem to be different, if not opposite from the effects of other negative emotions such as sadness or anxiety. They attribute this to the differences in appraisal of certainty between the different negative emotions. While anger is accompanied by a sense of certainty about the causes of the angering event, anxiety arises with a feeling of uncertainty about the cause of the event. This appraisal tendency leads to different processing depths. While emotions accompanied by a sense of certainty lead to more stereotyping and heuristic processing, emotions which are accompanied by a sense of uncertainty lead to more systematic, thorough processing [28, 29]. Therefore, the effect of arousal on cognition might not be a simple one, with higher arousal interfering more with cognitive performance, but rather an interaction of appraisal tendencies and arousal that lead to different arousal effects for different emotions.

Beyond the tendency of a shallow processing depth, anger is known to influence other cognitive processes such as decision-making and attention [30–33]. Hemenover and Zhang [30] found that anger activated a defensive optimism that caused participants to deemphasize the importance and impact of negative events. In studies from DeSteno and colleagues [34, 35], anger group participants rated angering events as more likely and found angry arguments to be more persuasive than sad arguments. Compared to sad people, angry people found dispositional attributions to be more likely and judged ambiguous events to be more likely caused by another person [36]. Similarly, the blame placed on a perpetrator increased with the increase of anger in participants [37]. Anger is also known to increase bias against outgroup members [38] and decrease trust in others [39]. Taken together, these results imply that anger influences cognitive processes and especially social decision-making significantly, usually in a direction that implies less willingness to take the other’s perspective.

To further study the relationship of state anger and social cognition, we conducted a series of three studies. All studies are based on a between group study design where we expected an increased state anger in our experimental group after the different anger induction procedures. We hypothesized that being in an angry state reduces empathy and interferes with ToM.

In Study 1, we used an autobiographical recall paradigm to induce anger in one group. We then employed an empathic accuracy paradigm [40–42], measuring the ability to accurately rate other´s affect based on an emotional narration and thus requiring both empathy and ToM. We hypothesized a reduction of empathic accuracy in the anger group. In Study 2 and 3 we used two real-life social interaction paradigms before conducting the EmpaToM paradigm [5], a paradigm assessing empathy and ToM with separate tasks. We predicted that both the sharing of others’ affect as well as compassion and ToM ability in angry participants is reduced. Additionally, we predicted an increase in confidence in anger group participants, as anger is known to increase certainty appraisals. In Study 2, we induced anger through the personal degradation by a supposed second participant (confederate) in the form of negative feedback. This paradigm has been used before and showed satisfying anger induction results [43, 44]. For Study 3, we developed a new anger induction paradigm. Our aim was to elicit anger without the personal devaluation of the participant to avoid inducing additional negative feelings such as shame or embarrassment. We therefore induced anger through the frustration of the participant by a confederate experimenter.

As the magnitude of effects of the anger induction on social cognition was unknown, we based the sample size in all three studies on other mood induction paradigms in the literature, opting for a sample of N = 40 to 50 [45–47]. We report the methods and results of all three studies as well as an additional analysis of the pooled data of Studies 2 and 3.

## Study 1: Autobiographical recall anger induction and its effect on empathy using an empathic accuracy task

### Study 1: Materials and methods

#### Participants

In *Study 1*, forty-six students of the Hebrew University of Jerusalem participated. One participant was excluded from further analysis due to a technical problem during the task. Analysis was conducted on the data of *N* = 45 participants (*n* = 25 women, *M*_*age*_ = 25.6, *SD*_*age*_ = 3.1, 21- to 34-years-old). Participants were randomly assigned to the control group (*n* = 23) or the experimental group (*n* = 22). Sample characteristics are summarized in Table 1. Participants gave written consent and were compensated with either course credit or 40NIS. Ethical approval was given by the Hebrew University of Jerusalem (NR ethics approval letter 12_17).

**Table 1:** Sample characteristics of all three studies.

Study	CG		EG	
	Gender	Age	Gender	Age
Study 1 (N = 45)	14f, 9m	25.74 (3.30)	11f, 11m	25.55 (3.04)
Study 2 (N = 49)	19f, 3m	21.18 (2.36)	19f, 8m	21.52 (2.62)
Study 3 (N = 46)	10f, 11m	22.38 (3.79)	10f, 15m	21.80 (3.04)

*Note*: Age is given in mean years with standard deviation in brackets; m = male, f = female¸ CG = control group, EG = experimental group.

#### Anger induction paradigm

We used an anger induction paradigm based on autobiographical recall to induce anger in the experimental group [18, 45, 48]. Participants were asked by the experimenter to recall a real-life situation in which they experienced anger, and to describe it in writing in detail, as vividly as possible, for five minutes, while sitting comfortably alone in the experiment room. Control group participants were asked to write about their daily routine for the same duration of time.

We measured anger with a momentary emotion questionnaire. The momentary emotion questionnaire contained 14 items in which participants rated on a 10 cm visual analog scale with the extremes “not at all” and “very much”. We focused on three items, asking them to rate their current state (e.g. anger, relaxation and shame).

#### Empathy measure

Participants underwent an empathic accuracy task [41, 42]. During the task, a fixation cross appeared (120 ms), followed by a short video clip (2–3 min) depicting a person telling a personal story (“target”). During the presentation of each video, participants were asked to continuously rate how positive or negative they believe the target felt when they told the story. Since we have previously collected these rating from the targets themselves, we could compute a correlation between targets’ and perceivers’ ratings, which produces a measure of empathic accuracy (EA). Participants were presented with four different videos, in counterbalanced and alternated order of either positive or negative content.

#### Procedure

After signing the consent form, participants were asked to fill out a sociodemographic questionnaire, followed by a momentary emotion questionnaire (T1). Then the anger induction paradigm was implemented, followed by the second emotion questionnaire (T2). After T2, participants performed the EA paradigm, and then repeated the emotion questionnaire for the third time (T3). A debriefing followed after the experiment. At home, participants filled out several personality questionnaires, which are not further considered in the current paper. These included the Hebrew version of the Interpersonal Reactivity Index [IRI, 49], and the TAS-20 alexithymia scale [50].

#### Statistical analysis

We analyzed the success of the anger induction as well as the performance in the empathic accuracy task. All analyses were conducted with IBM SPSS statistics 27.0.1.0 [51].

*Anger Induction*. To examine the success of the anger induction paradigm, we used change scores for the anger, relaxation and shame values of the emotion questionnaire as our outcome measures. As all three change variables were not normally distributed, we proceeded to compare group differences using Mann-Whitney-U tests for *AngerChange*, *RelaxationChange* and *ShameChange*.

*Empathic Accuracy*. To examine the effects of anger on empathic accuracy, we analyzed the results of the *EA scores* using a repeated measures ANOVA with a between-subject factor of group (experimental vs. control), and a within -subject factor of video valence. To exclude the possibility of a short-decaying effect for anger, we further ran the same analysis for the *EA scores* on the first two videos only (considering the first positive and first negative videos for each subject).

### Study 1: Results

#### Success of the anger induction manipulation

**Fig 1:**
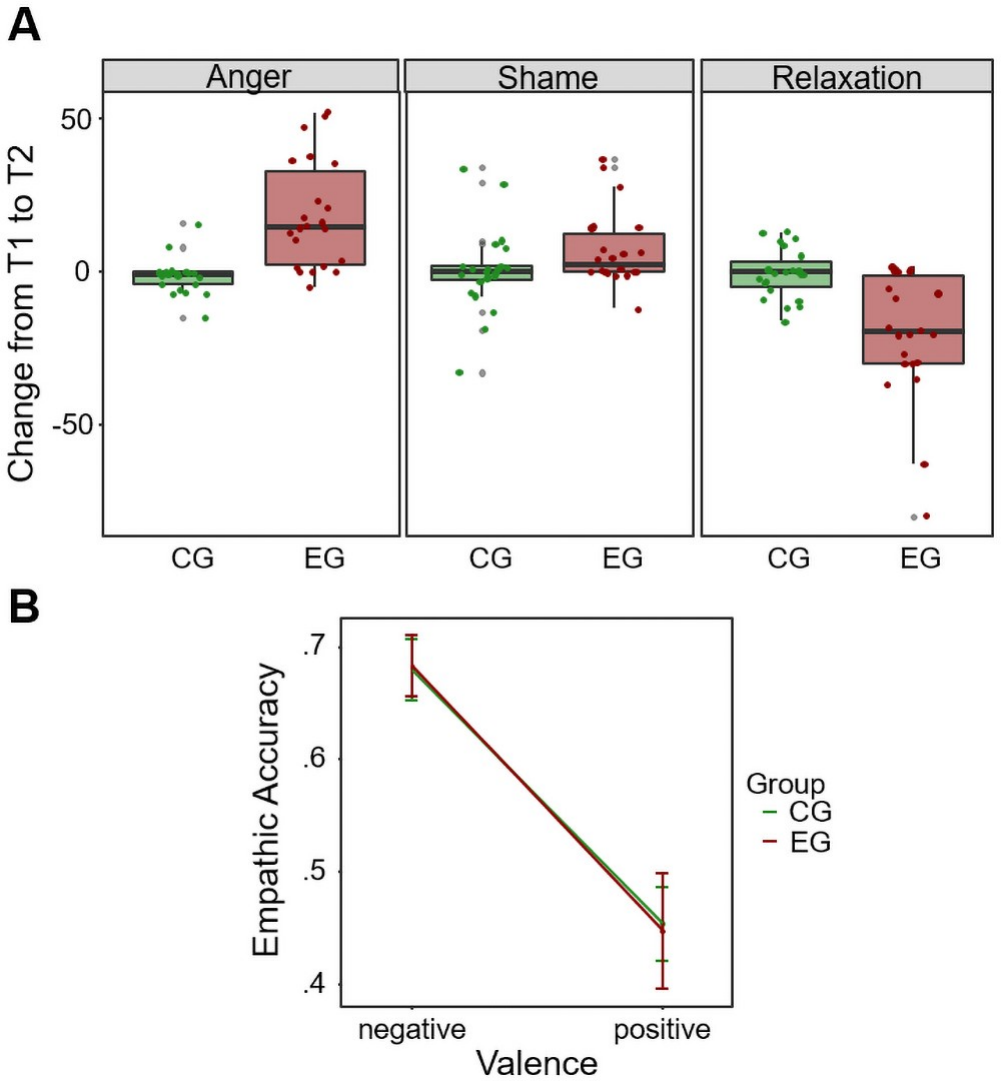
Results of anger induction and empathic accuracy paradigm in Study 1. A: Change in emotion ratings from pre to post anger induction in both groups (CG: control group, green bars; EG: experimental group, red bars). B: Averaged EA scores as a function of group (control–green; experimental–red) and valence of video. Scores are based on all trials.

On average, participants in the experimental group reported a larger increase in their *Anger* scores compared to those in the control group (*U* = 49.00, *z* = -4.68, *p* < .001, *r* = -.70, 95% CI [-.82, -.52]; Fig 1A). In addition, experimental group participants exhibited a significantly larger decline in *Relaxation* compared to control group participants (*U* = 106.00, *z* = -3.34, *p* = .001, *r* = -.50, 95% CI [-.66, -.21]). No group differences were observed in *Shame* ratings (*U* = 175.00, *z* = -1.76, *p* = .078, *r* = .26, 95% CI [-.03, .50]).

#### Results of empathic accuracy task

We observed a significant main effect of valence on *EA scores* (*F*(1,43) = 49.81, *p* < .001, *η*_*p*_^*2*^ = .54, 95% CI [.32, .67]), showing higher empathic accuracy for negative videos. However, there was no main effect of group on *EA scores* (*F*(1,41) = .002, *p* = .964, *η*_*p*_^*2*^ = .00, 95% CI [.00, .02]), and no interaction of valence X group (*F*(1,43) = .022, *p* = .882, *η*_*p*_^*2*^ = .00, 95% CI [.00, .07]) (Fig 1B). Similar results were found when taking into account the first two videos only, with no main effect of anger induction on *EA scores* (*F*(1,43) = .85, *p* = .361, *η*_*p*_^*2*^ = .02, 95% CI [.00, .16]), and no significant interaction (*F*(1,43) = 1.65, *p* = .205, *η*_*p*_^*2*^ = .04, 95% CI [.00, .19]).

### Study 1: Summary

In this study, we were able to induce anger in our experimental group as predicted and succeeded in distinctly angering participants without increasing similar negative emotions like shame. However, we did not observe the hypothesized reduction of empathic accuracy in anger group participants. This might be because the anger experienced by the participants was not strong enough to influence their empathic abilities in the task.

## Study 2: Negative feedback paradigm anger induction and its effect on empathy and ToM using the EmpaTom task

### Study 2: Materials and methods

#### Participants

Fifty-four students of the University of Lübeck were recruited using the university’s mailing list. As we used deception in this study, psychology students of higher semesters were not invited. Two participants were excluded from further analysis due to their familiarity with the EmpaToM paradigm and three participants expressed doubt about the cover story and were subsequently excluded. Data analysis was performed on *N* = 49 participants (*n* = 38 women, *M*_*age*_ = 21.4, *SD*_*age*_ = 2.5, 18- to 27-years-old). Participants were randomly assigned to the control group (*n* = 22) or the experimental group (*n* = 27). Sample characteristics are summarized in Table 1. Participants gave written consent and were compensated with either course credit or 8 €/h. Ethical approval was given by the University of Lübeck (AZ18-257).

#### Anger induction paradigm

An anger induction paradigm based on negative feedback was implemented [43, 44]. Participants were asked to pick one of three topics for an essay. They then wrote a short essay (maximum two pages) using information on the topic provided by the experimenter. Afterwards, participants were given an essay of a supposed second participant and were asked to rate this essay. Finally, participants received written feedback on their own essay by the supposed second participant. This feedback was manipulated: control group participants received neutral to positive feedback, while participants of the experimental group received negative feedback. The feedback consisted of the rating of the essay on five dimensions (10-step rating): unintelligent—intelligent, thought-provoking–boring, not logical–logical, irrational–rational, remarkable–mediocre. Additionally, there was some space for open commentary, in which both negative and neutral feedback included an evaluation of the introduction and the strength of arguments, for example “The introduction is confusing and doesn´t fit in with the rest of the text.” (negative) versus “The introduction is logical and fits in with the rest of the essay.” (neutral).

To measure the success of the anger induction paradigm, subjects had to fill out an emotion questionnaire. It was an ad-hoc created list of items, including the six state anger items of the German translation [52] of the State-Trait Anger Expression Inventory (STAXI) by Spielberger [14] and additional self-formulated items. The complete questionnaire consisted of 16 items forming four subscales. The subscale *Anger* included six items (e.g., I am angry.), *Relaxation* included five items (e.g., I am calm.) and two items measured *Shame* (e.g., I am ashamed.). Furthermore, there were three distractor items (I feel sad.; I am tired.; I feel hungry). A full list of items can be found in the (S1 File). All items were rated on a 4-factor Likert scale from 1 = “not at all” to 4 = “very much”. Cronbach’s alpha was calculated to assess the internal consistency of all subscales for both measurement times. The internal consistency of the *Anger* subscales at each time point was found to be satisfying, with Cronbach’s alpha for *Anger 1 α* = .79 and *Anger 2 α* = .82. Internal consistencies of the *Relaxation* subscales were not satisfying, with *Relaxation 1 α* = .60 and *Relaxation 2 α* = .68 and the inter-item correlations of item 11(I am dreamy.) were very low (*r* = -.25 to *r* = .40). This item was therefore excluded from the following analysis, resulting in *α* = .70 and *α* = .68 for this subscale. The two *Shame* items did not correlate (α = .19 and .30), we therefore used only the item asking explicitly for feeling ashamed in the following analyses.

#### Empathy and theory of mind measure

We implemented the EmpaToM, a paradigm developed by Kanske and colleagues [5]. The EmpaToM measures four main outcomes: empathy, compassion, ToM ability and confidence. Empathy is defined as feeling *with* the target person, i.e., sharing the others affect. Participants rate their own affect after watching a video of another person telling a personal story. If the rated affect matches the valence of the observed video, we interpret this as empathy. Compassion is defined as feeling positive emotions of kindness and care *for* the target person. Participants rate their own compassion after each video. ToM ability is defined as inferring the target’s mental state and operationalized as accuracy in answering ToM questions about the videos. Lastly, participants rate their own confidence in their answer. Additional to these variables, the EmpaToM allows us to study the distracting effects of emotion on cognitive functioning by directly comparing performance after emotional vs. neutral videos (see e.g. [53]). Kanske and colleagues [5] found that participants made less errors when answering ToM questions compared to nonToM questions, as well as making less errors after emotional videos than after neutral ones.

The detailed sequence of one trial was as follows: Participants were presented with a fixation cross (1–3 s) followed by a short video clip (~ 15 s) depicting an actor telling a personal story of an event in their live. This story was either emotionally negative (e.g., events that caused deep sadness, fear, or guilt, such as a death or illness, an accident or a betrayal) or neutral (e.g., events that did not carry any significant emotions, such as a meeting with friends, decluttering the garage, an anecdote from childhood or describing a hobby). These videos gave rise to either a ToM question (e.g. Anna thinks that…a) her brother fell in love with her best friend, and this is why he watches cartoon movies with her. b) her brother´s being in love entirely changes his taste in movies. c) her brother plans to also watch action movies with her best friend.) or a nonToM, fact-based question (e.g. It is correct that…a) Anna´s boyfriend was at the party. b) Anna has been to France before and has brought some red wine. c) Anna has been living with friends for quite a while.). This resulted in four categories of videos. The videos depicted 12 different actors, each recounting one story for each category, resulting in 48 videos in total. After each video, participants were asked to rate their own affect (negative to positive) and how much compassion they feel for the person in the video (none to very much) on a visual analog scale. Each question was presented for 4 seconds. After another fixation cross (1–3 s), participants were asked to answer either a ToM question or a nonToM question. They were presented with the three multiple choice response options and had a maximum of 14 seconds to select the answer. After another fixation cross (0–2 s), participants were asked to rate their confidence in the answer they just gave (uncertain to certain, 4 s). Then the next trial would follow. Overall, the paradigm took around 20 minutes.

#### Procedure

After written informed consent, participants were asked to fill out a sociodemographic questionnaire. To measure the success of the anger induction paradigm, participants had to fill out the emotion questionnaire right before (T1) and after the anger induction (T2). Once participants finished the second emotion questionnaire, we administered the EmpaToM. In the end, participants filled out several personality questionnaires, which are not further considered in the current paper. These included the trait anger scale of the STAXI [52], the German revised version of the Rosenberg Self-Esteem Scale [54] and the German translation of the Self-Description Questionnaire III [55]. A debriefing followed the administration of the questionnaires, where participants were asked what they believed the study to be about. Through additional follow up questions, it was determined, whether the participant fell for the deception.

#### Statistical analyses

We analyzed the success of the anger induction as well as the performance in the EmpaToM paradigm. All analyses were conducted in R version 3.6.3 [56].

*Anger induction*. To examine the success of the anger induction paradigm, we used change scores for the anger, relaxation, and shame values of the emotion questionnaire as our outcome measures. As all three change variables were not normally distributed, we compared group differences using Mann-Whitney-U tests for *AngerChange*, *RelaxationChange* and *ShameChange*.

*EmpaToM*. For the EmpaToM, mean scores were calculated for the affect rating (scale from -3 to 3), compassion rating (scale from 0 to 6) and confidence rating (scale from 1 to 6). As missing values were extremely rare in these variables, means were calculated ignoring missing values. For the accuracy variable, both an incorrect answer and no answer was coded as a wrong answer and accuracy was calculated as the quota of correct answers for each condition (scale from 0 to 1).

Group differences in the four outcome variables were analyzed by means of a mixed design ANOVA with the within-subject factors emotionality of video (emotionally negative versus neutral) and ToM requirement of the question (ToM versus nonToM). Where appropriate, we report both partial eta squared and generalized eta squared to allow for the comparison of our effects to studies with the same experimental design as well as studies with other experimental designs [57]. Interaction effects in the ANOVA were followed by post hoc analyses, allowing us to better asses our hypotheses. We report simple main effects with Bonferroni correction for the effect of group on affect and compassion ratings of emotionally negative videos, as well as the effect of group on accuracy in ToM questions. Additionally, we report the simple main effects of emotionality and ToM requirement on accuracy in both groups, in order to compare the performance of our groups to that of previous studies using the EmpaToM (see [5]). We repeated this analysis adding time as a factor, to investigate if the effect of the anger induction changes and possibly subsides over time. However, as we did not find any evidence that performance changed in a systematic way over the course of the experiment, we report these results in the (S4 File).

### Study 2: Results

#### Success of the anger induction manipulation

**Fig 2:**
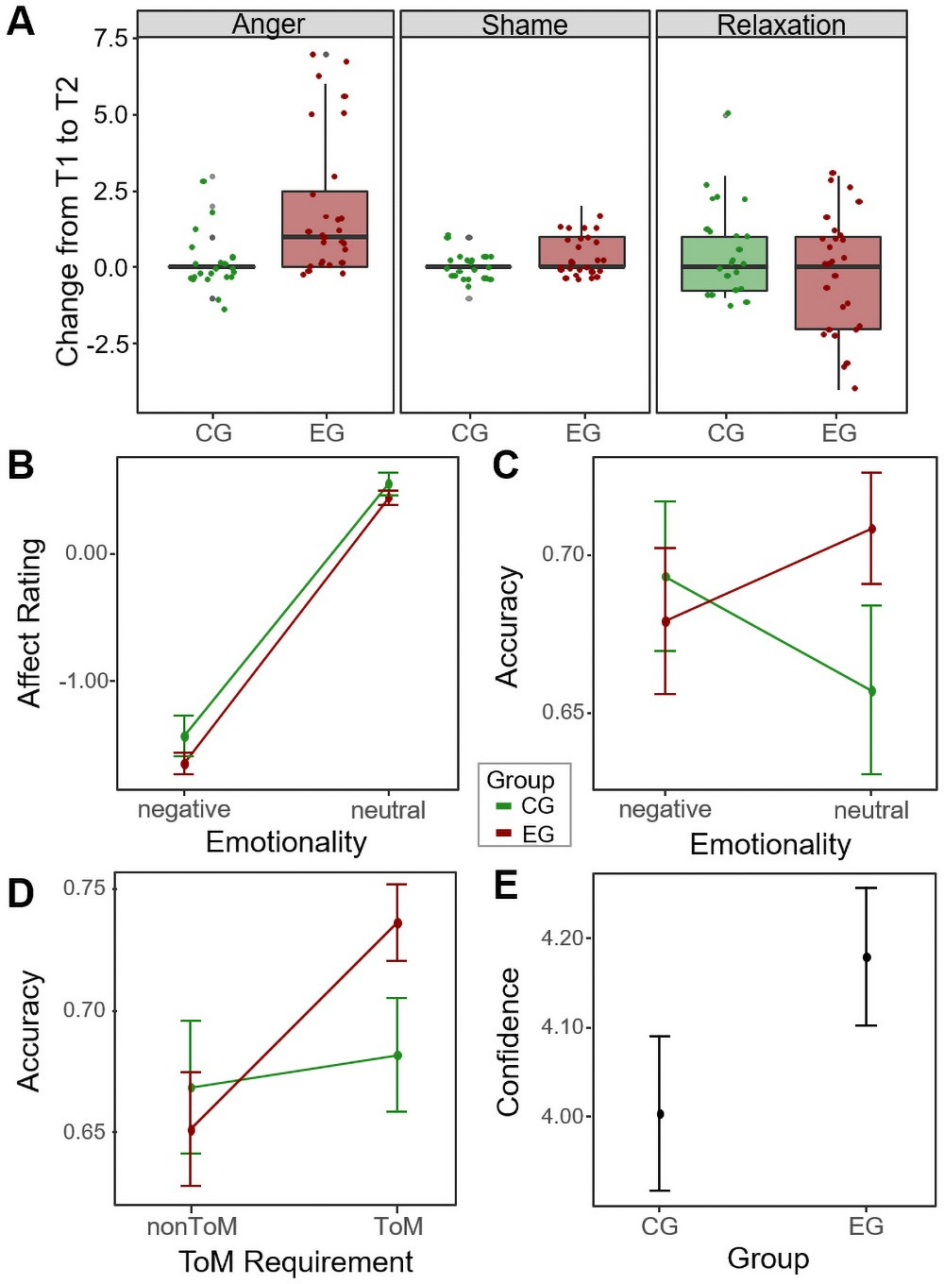
Results of the anger induction based on negative feedback and the EmpaToM paradigm in Study 2. A: Change in emotion ratings from pre to post anger induction in both groups. B: Effect of group and emotionality of video on affect ratings in the EmpaToM. C: Effect of group and emotionality of video on accuracy in the EmpaToM. D: Effect of group and ToM requirement of question on accuracy in the EmpaToM. E: Group effect on confidence in EmpaToM.

Ratings of anger increased significantly more in the experimental group than the control group (*U* = 467.50, *z* = 3.67, *p* < .001, *r* = .52, 95% CI [.28, .70]; Fig 2A). Participants of the experimental group reported increased shame relative to the control group (*U* = 389.50, *z* = 2.42, *p* = .016, *r* = .35, 95% CI [.08, .57]). Relaxation on the other hand did not show a significant group effect (*U* = 239.50, *z* = -1.17, *p* = .240. *r* = -.17, 95% CI [-.43, .12]).

#### Results of EmpaToM Task

A 2 (group: angry vs. neutral) x 2 (emotionality of video: negative vs. neutral) x 2 (ToM requirement: ToM vs. nonToM) mixed design ANOVA was run on all four outcome variables of the EmpaToM: *affect rating*, *compassion rating*, *accuracy* and *confidence rating*. Here, we will only report relevant results concerning the effects of the anger induction on the affect and compassion ratings as well as the accuracy in answering the questions and the confidence in those answers. However, we were able to replicate the main task findings of Kanske and colleagues [5] and all results can be found in the (S4 File).

Detailed results can be found in Table 2. Anger group participants did not differ in their *affect* compared to control group participants (Fig 2B). Similarly, an analysis of the simple main effect of group after emotional videos revealed no effect. A similar picture emerged for the *compassion rating*. Participants in the anger group did not differ in their compassion. Additionally, when considering only emotional videos, there was no difference in compassion ratings between both groups.

**Table 2:** ANOVA results and post hoc analyses of EmpaToM outcomes in Study 2.

**Affect Rating**								
**2x2x2 Anova**								
Predictor		*df*_*Num*_	*df*_*Den*_	*F*	*p*	η ^2^_p_	*95% CI*	η^2^_g_
Group		1	47	1.13	.294	.02	[.00, .16]	.01
Group x Emotionality		1	47	0.20	.660	.00	[.00, .10]	.00
**Simple main effect**								
Emotionality	Effect	*df*_*Num*_	*df*_*Den*_	*F*	*p*	η ^2^_p_	*95% CI*	
negative	Group	1	47	0.80	.377	.02	.00-.10	
**Compassion Rating**								
**2x2x2 Anova**								
Predictor		*df*_*Num*_	*df*_*Den*_	*F*	*p*	η ^2^_p_	*95% CI*	η^2^_g_
Group		1	47	0.07	.791	.00	[.00, .08]	.00
Group x Emotionality		1	47	0.05	.825	.00	[.00, .07]	.00
**Simple main effect**								
Emotionality	Effect	*df*_*Num*_	*df*_*Den*_	*F*	*p*	η ^2^_p_	*95% CI*	
negative	Group	1	47	0.01	.909	.00	[.00, .05]	
**Accuracy**								
**2x2x2 Anova**								
Predictor		*df*_*Num*_	*df*_*Den*_	*F*	*p*	η ^2^_p_	*95% CI*	η^2^_g_
Group		1	47	0.41	.526	.01	[.00, .12]	.00
Group x Emotionality		1	47	4.14	.048	.08	[.00, .25]	.01
Group x ToM Requirement		1	47	2.44	.125	.05	[.00, .20]	.01
**Simple main effects**								
Group	Effect	*df*_*Num*_	*df*_*Den*_	*F*	*p*	η ^2^_p_	*95% CI*	η^2^_g_
EG	Emotionality	1	26	2.39	.134	.08	[.00, .31]	.02
CG	Emotionality	1	21	1.79	.195	.08	[.00, .33]	.02
EG	ToM Requirement	1	26	7.09	.013	.21	[.01, .44]	.11
CG	ToM Requirement	1	21	0.17	.686	.01	[.00, .19]	.00
ToM Requirement	Effect	*df*_*Num*_	*df*_*Den*_	*F*	*p*	η ^2^_p_	*95% CI*	
ToM questions	Group	1	47	3.11	.08	.06	[.00, .22]	
**Confidence Rating**								
**2x2x2 Anova**								
Predictor		*df*_*Num*_	*df*_*Den*_	*F*	*p*	η ^2^_p_	*95% CI*	η^2^_g_
Group		1	47	0.86	.357	.02	[.00, .15]	.01

*Note*. *df*_*Num*_ indicates degrees of freedom numerator. *df*_*Den*_ indicates degrees of freedom denominator. η ^2^_p_ indicates partial eta-squared. η^2^_g_ indicates generalized eta-squared.

Anger group participants were not generally less *accurate* in answering the questions; however, there was a significant interaction effect of emotionality and group (Fig 2C). The accuracy values suggested that anger group participants were more accurate in answering questions after neutral videos than after emotionally negative videos, whereas controls showed the inverse effect, but none of these simple effects reached significance. The interaction of ToM requirement and group did not yield significance (Fig 2D), but anger group participants were more accurate after ToM questions than after factual questions, while control participants’ accuracy was not influenced by the type of question. Considering only ToM questions, anger group participants were more accurate than controls, but this was not significant.

Finally, anger group participants did not differ in their confidence ratings (Fig 2E), nor did any interaction effects with group reach significance.

### Study 2: Summary

The results show that the anger induction was successful in the experimental group, while no increase of anger was observed in the control group. Shame increased in the experimental group as well, indicating that the paradigm was not able to induce discrete anger. There was no effect on levels of relaxation between the groups.

In the EmpaToM, we found the expected and previously shown task effects, speaking for the effectiveness of the paradigm to measure empathy, compassion and ToM. Regarding the influence of anger, we did not find an effect of anger on the sharing of affect or compassion in general nor after emotional videos, indicating that both forms of empathic responses in our groups were not altered by their emotional state. Furthermore, we did not observe a general decline in ToM performance in anger group participants. On the contrary, the accuracy of participants in the anger group was dependent on the type of question. They were more accurate in answering ToM questions rather than factual ones, while the type of question did not influence control participants. Furthermore, anger group participants performed worse after emotionally negative videos, while control participants struggled more with the questions following neutral videos. Previous studies with the EmpaToM found similar effects as in our control group, suggesting that answering questions is easier after emotional videos [5]. Therefore, our results indicate that being in an angry state alters the usual preference for emotional videos.

## Study 3: Frustration paradigm anger induction and its effect on empathy and ToM using the EmpaToM task

### Study 3: Materials and methods

#### Participants

Fifty-seven students of the University of Lübeck participated in the study. As we used deception in this study, psychology students of higher semesters were not invited. Eleven participants were excluded from further analysis due to them not believing the paradigm (6), being too enraged to continue the experiment (1), having severe problems with vision (1), quitting the experiment early (1) or showing heightened anger levels before the anger induction (>2SD) (2). Analysis was conducted on the data of *N* = 46 participants (*n* = 20 women, *M*_*age*_ = 22.1, *SD*_*age*_ = 3.4, 18- to 30-years-old). Participants were randomly assigned to the control group (*n* = 21) or the experimental group (*n* = 25). Participants gave written consent and were compensated with either course credit or 8 €/h. Ethical approval was given by the University of Lübeck.

#### Anger induction paradigm

We developed an anger induction paradigm based on frustration to induce anger in the experimental group. Participants were asked by a confederate experimenter to fill out a tedious registration form as part of the registration process of the institute. Once participants finished the registration form, the confederate would find a minor mistake, tear the sheet apart and order the participant to fill out the form again. This process was repeated another time. In total, experimental group participants had to fill out the form three times. Control group participants simply filled the form out once. It is important to note that this type of anger induction did not allow for the experimenter to be blinded to the groups.

To measure anger, participants filled out the same emotion questionnaire as in Study 2. The only changes were a slightly altered scale. All items were rated on a 10 cm visual analog scale with the extremes “not at all” and “very much”. We chose the visual analog scale to increase sensitivity for subtle intraindividual fluctuations in subject´s emotional state after the anger induction. The results of the internal consistency were similar to Study 2. We therefore excluded the same items from the *Relaxation* and the *Shame* subscale respectively.

#### Empathy measure

We implemented the EmpaToM, the same paradigm as in Study 2 (see Study 2 Methods).

#### Procedure

The procedure was similar to Study 2. Participants filled out a sociodemographic questionnaire, followed by the emotion questionnaire. Then the anger induction paradigm was implemented, followed by the second emotion questionnaire. Then, participants performed in the EmpaToM paradigm.

#### Statistical analyses

We analyzed the success of the anger induction as well as the performance in the EmpaToM paradigm in the exact same manner as we did in Study 2. All analyses were conducted in R version 3.6.3 [56].

### Study 3: Results

#### Success of the anger induction manipulation

**Fig 3:**
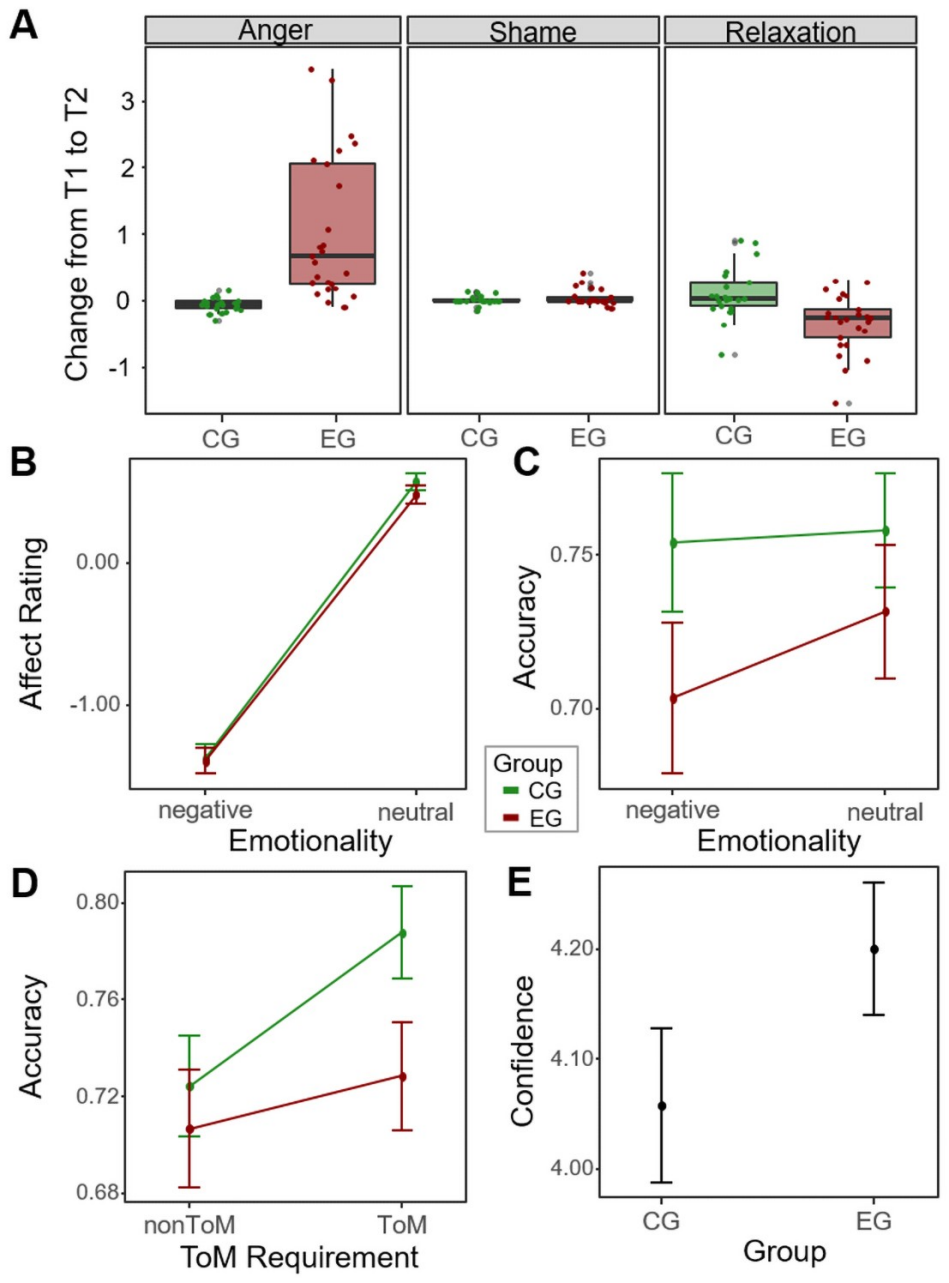
Results of the anger induction based on frustration and the EmpaToM paradigm in Study 3. A: Change in emotion ratings from pre to post anger induction in both groups B: Effect of group and emotionality of video on affect ratings in the EmpaToM. C: Effect of group and emotionality of video on accuracy in the EmpaToM. D: Effect of group and ToM requirement of question on accuracy in the EmpaToM. E: Group effect on confidence in EmpaToM.

Participants of the experimental group reported a higher increase of *Anger* than those in the control group (*U* = 501.00, *z* = 5.26, *p* < .001, *r* = .77, 95% CI [.62, .87]; Fig 3A). Simultaneously, experimental group participants exhibited a significantly higher decline in *Relaxation* than control group participants did (*U* = 109.50, *z* = -3.38, *p* = .001, *r* = -.50, 95% CI [-.69, -.25]). No group difference was observed in *Shame* ratings compared to the control group (*U* = 284.00, *z* = 0.48, *p* = .63, *r* = .07, 95% CI [-.23, .35]).

#### Results of EmpaToM Task

A 2 (group: angry vs. neutral) x 2 (emotionality of video: negative vs. neutral) x 2 (ToM requirement: ToM vs. nonToM) mixed design ANOVA was run on all four outcome variables of the EmpaToM: *affect rating*, *compassion rating*, *accuracy* and *confidence rating*. Here, we will only report relevant results. As in Study 2, we were able to replicate the main task findings of Kanske and colleagues [5] and all results can be found in the (S6 File).

Detailed results can be found in Table 3. There was no main effect of anger induction on *affect*, nor a significant interaction between group and emotionality (Fig 3B). Additionally, we did not find a group difference in affect ratings when considering only emotional videos. Similarly, there was no main effect of group on *compassion*, nor did the interaction with emotionality reach significance. Both groups reported similar compassion after emotional videos, replicating the results of Study 2.

**Table 3:** ANOVA results and post hoc analyses of EmpaToM outcomes in Study 3.

**Affect Rating**								
**2x2x2 Anova**								
Predictor		*df*_*Num*_	*df*_*Den*_	*F*	*p*	η ^2^_p_	*95% CI*	η^2^_g_
Group		1	44	0.33	.568	.00	[.00, .12]	.00
Group x Emotionality		1	44	0.09	.769	.00	[.00, .09]	.00
**Simple main effect**								
Emotionality	Effect	*df*_*Num*_	*df*_*Den*_	*F*	*p*	η ^2^_p_	*95% CI*	
negative	Group	1	44	0.01	.939	.00	[.00, .05]	
**Compassion Rating**								
**2x2x2 Anova**								
Predictor		*df*_*Num*_	*df*_*Den*_	*F*	*p*	η ^2^_p_	*95% CI*	η^2^_g_
Group		1	44	1.85	.180	.04	[.00, .20]	.02
Group x Emotionality		1	44	1.59	.213	.04	[.00, .18]	.01
**Simple main effect**								
Emotionality	Effect	*df*_*Num*_	*df*_*Den*_	*F*	*p*	η ^2^_p_	*95% CI*	
negative	Group	1	44	0.09	.765	.00	[.00, .09]	
**Accuracy**								
**2x2x2 Anova**								
Predictor		*df*_*Num*_	*df*_*Den*_	*F*	*p*	η ^2^_p_	*95% CI*	η^2^_g_
Group		1	44	1.53	.223	.03	[.00, .18]	.02
Group x Emotionality		1	44	0.57	.456	.01	[.00, .14]	.00
Group x ToM Requirement		1	44	1.29	.262	.03	[.00, .17]	.01
**Simple main effects**								
Group	Effect	*df*_*Num*_	*df*_*Den*_	*F*	*p*	η ^2^_p_	*95% CI*	η^2^_g_
EG	Emotionality	1	24	1.39	.250	.06	[.00, .28]	.01
CG	Emotionality	1	20	0.04	.850	.00	[.00, .14]	.00
EG	ToM Requirement	1	24	0.61	.444	.03	[.00, .22]	.00
CG	ToM Requirement	1	20	7.91	.011	.28	[.02, .52]	.10
ToM Requirement	Effect	*df*_*Num*_	*df*_*Den*_	*F*	*p*	η ^2^_p_	*95% CI*	
ToM questions	Group	1	44	2.98	.091	.06	[.00, .23]	
**Confidence Rating**								
**2x2x2 Anova**								
Predictor		*df*_*Num*_	*df*_*Den*_	*F*	*p*	η ^2^_p_	*95% CI*	η^2^_g_
Group		1	44	0.94	.336	.02	[.00, .16]	.01

*Note*. *df*_*Num*_ indicates degrees of freedom numerator. *df*_*Den*_ indicates degrees of freedom denominator. η ^2^_p_ indicates partial eta-squared. η^2^_g_ indicates generalized eta-squared.

Looking at *accuracy*, anger group participants were slightly less accurate than controls, but this main effect of group was not significant. Contrary to Study 2, we did not find a significant interaction of emotionality and group (Fig 3C). An analysis of simple main effects showed that anger group participants were nominally more accurate in responding to questions after neutral videos than after emotionally negative videos, whereas controls showed no such effect. We also did not find an interaction of ToM requirement and group (Fig 3D). Unlike in Study 2, anger group participants were not influenced by the type of questions, while control participants were more accurate after ToM questions. The simple main effect of group when only looking at ToM questions showed that anger group participants were slightly less accurate than controls in answering ToM questions.

Similar to Study 2, anger group participants did not differ in their confidence ratings (Fig 3E), nor did any interaction effects with group reach significance.

### Study 3: Summary

Analysis of the data showed that the anger induction was successful in the experimental group, while no increase of anger was observed in the control group. Shame did not increase in the experimental group, indicating that our developed paradigm is able to induce discrete anger.

Similar to the previous study, we did not find an effect of anger on the sharing of affect or compassion in general nor after emotional videos, indicating that empathy in our groups was not altered by their emotional state. Furthermore, we did not observe a general decline in accuracy in our anger group participants. However, when only looking at ToM questions, participants of the anger group performed slightly worse than the control group, indicating that anger did impair their perspective taking. Contrary to the previous study, we did not find that emotionality of the videos influenced our groups in different ways. However, we observed the same trend as previously, suggesting that anger group participants are better at answering questions after neutral videos.

## Analysis of pooled data: Study 2 and Study 3

As the anger induction effects were comparable between Study 2 and 3 and to increase the power for our analyses, we also analyzed the pooled data of both studies. We therefore did the same mixed ANOVA on the EmpaToM data as in the previous studies but included *type of anger induction* as a between group variable. Simulation based power analysis was done using the R function Superpower [58]. Effect sizes were estimated based on a previous study comparing men with a history of legally relevant aggressive behavior with controls [3], as we determined this set up to be the closest one to ours. We thus estimated medium effects for differences in empathy measures and small effects for differences in ToM measures. The power analysis yielded sufficient power (above 80%) for detecting medium effects in the hypothesized interaction of emotionality and group on our empathy measures (*affect* and *compassion*), and sufficient power for detecting large effects in the interaction of ToM requirement and group on our ToM measure (*accuracy*).

A 2 (group: angry vs. neutral) x 2 (type of anger induction: negative feedback vs. frustration) x 2 (emotionality of video: negative vs. neutral) x 2 (ToM requirement: ToM vs. nonToM) mixed design ANOVA was run on all four outcome variables of the EmpaToM: *affect rating*, *compassion rating*, *accuracy* and *confidence rating*. No relevant effect was influenced by the type of anger induction; thus, we will only report the effects regarding our hypotheses. Full results can be found in the (S7 File).

Detailed results are in Table 4. There was no main effect of anger induction on *affect*, nor a significant interaction between group and emotionality. Additionally, we did not find a group difference in affect ratings when considering only emotional videos. Similarly, there was no main effect of group on *compassion*, nor did the interaction with emotionality reach significance. Both groups reported similar compassion after emotional videos.

**Table 4:** ANOVA results and post hoc analyses of EmpaToM outcomes in pooled data.

**Affect Rating**								
**2x2x2 Anova**								
Predictor		*df*_*Num*_	*df*_*Den*_	*F*	*p*	η ^2^_p_	*95% CI*	η^2^_g_
Group		1	91	1.41	.238	.02	[.00, .10]	.00
Group x Emotionality		1	91	0.01	.928	.00	[.00, .05]	.00
**Simple main effect**								
Emotionality	Effect	*df*_*Num*_	*df*_*Den*_	*F*	*p*	η ^2^_p_	*95% CI*	
negative	Group	1	93	0.59	.443	.01	[.00, .07]	
**Compassion Rating**								
**2x2x2 Anova**								
Predictor		*df*_*Num*_	*df*_*Den*_	*F*	*p*	η ^2^_p_	*95% CI*	η^2^_g_
Group		1	91	0.47	.493	.00	[.00, .07]	.00
Group x Emotionality		1	91	0.64	.425	.01	[.00, .08]	.00
**Simple main effect**								
Emotionality	Effect	*df*_*Num*_	*df*_*Den*_	*F*	*p*	η ^2^_p_	*95% CI*	
negative	Group	1	93	0.01	.935	.00	[.00, .02]	
**Accuracy**								
**2x2x2 Anova**								
Predictor		*df*_*Num*_	*df*_*Den*_	*F*	*p*	η ^2^_p_	*95% CI*	η^2^_g_
Group		1	91	0.22	.639	.00	[.00, .06]	.00
Group x Emotionality		1	91	3.86	.052	.04	[.00, .14]	.01
Group x ToM Requirement		1	91	0.25	.616	.00	[.00, .06]	.00
**Simple main effects**								
Group	Effect	*df*_*Num*_	*df*_*Den*_	*F*	*p*	η ^2^_p_	*95% CI*	η^2^_g_
EG	Emotionality	1	51	3.68	.061	.07	[.00, .22]	.02
CG	Emotionality	1	42	0.92	.343	.02	[.00, .14]	.01
EG	ToM Requirement	1	51	6.40	.015	.11	[.00, .28]	.04
CG	ToM Requirement	1	42	3.56	.066	.08	[.00, .25]	.02
ToM Requirement	Effect	*df*_*Num*_	*df*_*Den*_	*F*	*p*	η ^2^_p_	*95% CI*	
ToM questions	Group	1	93	0.00	.962	.00	[.00, .01]	
**Confidence Rating**								
**2x2x2 Anova**								
Predictor		*df*_*Num*_	*df*_*Den*_	*F*	*p*	η ^2^_p_	*95% CI*	η^2^_g_
Group		1	91	1.74	.191	.02	[.00, .10]	.00

*Note*. *df*_*Num*_ indicates degrees of freedom numerator. *df*_*Den*_ indicates degrees of freedom denominator. η ^2^_p_ indicates partial eta-squared. η^2^_g_ indicates generalized eta-squared.

Looking at *accuracy*, anger group participants were slightly less accurate than controls but this main effect of group was not significant. We did find a marginally significant interaction of emotionality and group. The anger group participants were slightly, but not significantly more accurate after neutral videos than after emotionally negative videos, while the control´s accuracy was not influenced by emotionality. We did not find an interaction of ToM requirement and group. However, anger group participants were influenced by the type of question, while control participants were not. Looking only at ToM questions, anger group participants were not less accurate than controls.

Anger group participants did not differ in their confidence ratings, nor did any interaction effects with group reach significance.

### Pooled data: Summary

Pooling the data of studies 2 and 3, we did not find an effect of anger on shared affect or compassion in general nor after emotional videos, indicating that empathy in our anger group participants was not altered by their emotional state. Furthermore, we did not observe a general decline in accuracy in our anger group participants, nor did they perform significantly worse in ToM questions. However, the accuracy of participants in the anger group was dependent on the type of question. They were more accurate in answering ToM questions rather than factual ones, while the type of question did not influence our control participants. Furthermore, anger group participants performed slightly worse after emotionally negative videos. However, the interaction with the factor group was not significant for both emotionality and ToM requirement.

## Discussion

With the present study, we aimed to investigate the influence of state anger on behavioral measures of empathy and ToM. To capture different levels of anger states we implemented already established anger induction paradigms and developed a novel interactive anger induction paradigm. After successful anger induction, we examined its impact on subsequent measures of empathy and ToM. Contrary to our hypotheses, an increase in state anger did not affect empathic accuracy, sharing of affect, compassion, ToM performance or confidence ratings. In the following, we will first discuss the anger induction paradigms and then discuss the lack of effects of the anger induction on empathy and theory of mind.

### Anger induction paradigms

All anger induction paradigms, autobiographical recall, negative feedback and frustration successfully induced anger. When comparing effect sizes, the autobiographical recall and frustration paradigms outperformed the negative feedback paradigm with large effect sizes for the group differences in anger and relaxation ratings. A recent meta-analysis found a similar average of effect sizes in anger induction studies and also underlined that an increase of negative affect is usually accompanied by a decrease of positive effect with a similar magnitude [59]. However, as participants in the autobiographical recall paradigm are explicitly instructed to recall angering events and are thus not as blind to the induction, the good results might partially be due to demand characteristics [59].

Furthermore, the negative feedback paradigm also led to heightened feelings of shame, which was not the case for the other two paradigms. Focusing on the interactive paradigms, the more specific effect on anger in the frustration paradigm can be explained with the paradigm not degrading the self-worth of the participant. Additionally, the anger effect might have been stronger, because participants were face to face with the anger-inducing person, rather than receiving written feedback. This is in line with previous research, showing that personal interaction leads to stronger anger induction than written feedback, possibly making it harder for the participant to disengage from the encounter [23]. A downside of the new induction method is its lower believability. More people expressed doubt over its cover story than it was the case for the negative feedback paradigm. Suspicion in deception experiments has been shown to alter participants’ behavior. This suspicion can be facilitated by prior experience with deceptive experiments [60]. To counteract this effect, we excluded psychology students in their second year or higher. However, as all participants were students with ample opportunity to participate in studies, we cannot rule out prior contact with studies featuring deception. This might have made it more difficult to induce strong anger in this group. Thus, the paradigm might be even more successful in a general population with less prior experience in psychological experiments.

Furthermore, we only used a self-report measurement for anger. These measures have limitations like a social desirability bias, demand effects, the ability of participants to correctly label and quantify their emotions and their sensitivity to faint fluctuations [23, 61]. It might therefore be instructive to combine these measures with psychophysiological measures in future studies.

In summary, all paradigms were successful in eliciting anger in the target participants. Advantages of the new paradigm based on frustration are the ecological validity and no simultaneous increase of shame. It should be noted however that although all paradigms clearly induced anger on average, the effect was quite variable across participants with a considerable number of individuals not showing any change in negative affect at all. While this is advantageous for studies on interindividual variation, it reduces the power to detect effects of anger induction on the group level. It might be interesting to perform the reported analysis including only participants with a high increase of anger in future studies with bigger sample sizes.

### Influence of anger on empathy and ToM

Contrary to our hypothesis, we did not find any general impairment by anger in empathic accuracy, empathy and ToM. These results seem to contradict previous studies that found a connection between empathy and anger. However, these studies looked at anger and empathy as personality traits [1, 2]. In the current study, we examined the effect of state anger on state social cognition, which was found to be unrelated in our experiments.

There are different explanations as to why the anger induction did not impact performance in the EA and EmpaToM paradigms. First, this might be due to emotions elicited by these video-based paradigms themselves, which might have been more intense than the anger induction in the beginning. Indeed, the EA and EmpaToM paradigms are quite similar to typical video-based emotion induction paradigms. Second, the anger induction might have been too weak and transient. Although the effect was statistically strong, several participants did not show an anger induction effect or reported only weak changes in anger. Also, it might be that our behavioral measures of empathy and ToM were not sensitive enough to detect subtle changes in the participants’ response to others’ emotions. Furthermore, our studies with relatively small sample sizes only allowed for the detection of medium to large effect sizes. We improved the power by pooling the data from Studies 2 and 3 but might have still missed small effects. Additionally, the small sample size did not allow for controlling possible covariates such as gender or in/outgroup status between participants and the confederate or the observed people in the videos.

Our data thus questions whether anger status has a general effect on understanding other’s emotions and cognition. If there are effects of anger on empathy, the anger induction was not strong enough to elicit them. It might also be that the effects are more affect-specific such that anger status helps one to resonate with other’s anger but not with other’s fear or sadness. The heterogeneity of our video stimuli does not allow for such specific analyses, but future studies should test more specifically the effects on understanding other’s sadness or anger. Future studies might also explore underlying physiological and brain activation patterns of empathic experience and how these are modulated by state anger, similar to previous studies of the impact of affective states on empathy and ToM [25, 62] as these measures might be more sensitive to detect anger effects.

Lastly, it may be that anger, being an emotion that is often expressed towards someone (person or group) may influence empathy towards that person or group only. Future experiments can test such a prediction. For example, if the ‘target’ figure in an empathic accuracy task is the confederate that just made you angry.

While we did not find a general effect of the anger induction on answering questions about the videos, anger group participants were better at answering questions after neutral videos compared to emotional videos. This differed from the behavior of the control participants, as well as from participants in previous studies using the EmpaToM [5, 63]. Participants across these EmpaToM studies were better at answering questions about emotional videos, which fits with memory research showing that emotional content is better remembered than neutral content [64]. This is explained with both memory-encoding factors such as attention as well as memory consolidation. As participants are asked about the videos immediately after watching them, our finding of an altered emotionality effect on accuracy might indicate that state anger alters the attentional bias for emotional information. It is important to highlight that this interaction was only significant in Study 2 but exhibited the same trend in Study 3. Similarly, we were not able to find a general decline in performance when only looking at ToM questions. On the contrary, similar to previous studies with the EmpaToM [5] our participants consistently performed better in ToM questions than factual questions. In the pooled data, this effect was even more pronounced in our angry subgroup. Thus, we were not able to replicate the findings of Yip and Schweitzer [26], who found reduced perspective-taking in non-emotional tasks in their angry participants. However, as they point out, their effect was mediated by the arousal elicited by the anger induction. Thus, our anger induction might have induced less arousal as would be necessary to impair ToM.

Contrary to our hypothesis, anger group participants were only nominally more confident about their responses. At first glance, this seems to contradict previous research, arguing that anger compared to other negative emotions increases perceived control and certainty [32]. However, the studies finding these effects focused on either the evaluation of future events or the attribution of blame or causality in past events [65]. Our results indicate that the feeling of certainty when being moderately angry does not translate to a significant increase of confidence in one´s own judgment about other’s current mental states.

## Conclusions

To summarize, we were able to validate a new interactive anger-induction paradigm based on the frustration of participants by goal blocking. We found no direct effect of anger on empathy in the empathic accuracy task, nor on ToM performance and empathy in the EmpaToM. However, there was a trend effect of anger on accuracy, indicating that anger group participants lose the natural preference for emotional content observed normally in the EmpaToM. Furthermore, anger group participants were consistently better at answering ToM questions compared to factual ones. While the results did not corroborate a direct effect of mild state anger on either empathy or theory-of-mind, it provides a starting point for further research into this topic. Future studies should assess, whether stronger anger (rage) or other emotions influence empathy and ToM directly or indirectly via attention and memory processes. Study designs should include physiological measures of anger and neural measures of empathy and ToM.

## Supporting information

S1 File Items of the emotion questionnaire used in Studies 2 and 3. (PDF)

S2 File Behavioral results of empathic accuracy paradigm in Study 1. (PDF)

S3 File Behavioral results of EmpaToM in Study 2. (PDF)

S4 File Task Effects in EmpaToM Study 2: The reviewer. (PDF)

S5 File Behavioral results of EmpaToM in Study 3. (PDF)

S6 File Task Effects in EmpaToM Study 3: The officer. (PDF)

S7 File Task Effects in EmpaToM: Pooled data of Studies 2 and 3. (PDF)
