# Supplementary material for: The influence of anger on empathy and theory of mind
Source: PLoS One. 2021 Jul 29;16(7):e0255068. doi: 10.1371/journal.pone.0255068 (PMC8321371; doi:10.1371/journal.pone.0255068)
Supplement: S1 File — (PDF) [file pone.0255068.s001.pdf]

S1 File. Items of the emotion questionnaire used in Studies 2 and 3

- I feel relaxed.
- I am furious.
- I feel sad.
- I feel stressed.
- I feel hungry.
- I am nervous.
- I am mad.
- I am calm.
- I feel enraged.
- I feel dreamy.
- I am proud.
- I am angry.
- I am tired.
- I am furious.
- I could go through the roof.
- I feel ashamed.
