# Supplementary material for: The influence of anger on empathy and theory of mind
Source: PLoS One. 2021 Jul 29;16(7):e0255068. doi: 10.1371/journal.pone.0255068 (PMC8321371; doi:10.1371/journal.pone.0255068)
Supplement: S2 File — (PDF) [file pone.0255068.s002.pdf]

S2 File. Behavioral results of empathic accuracy paradigm in

Study 1 Table 1

Group	CG				EG			
Valence	positive		negative		positive		negative	
	m	sd	m	sd	m	sd	m	sd
EA score	0.45	0.16	0.68	0.13	0.45	0.24	0.68	0.13
