# Supplementary material for: The influence of anger on empathy and theory of mind
Source: PLoS One. 2021 Jul 29;16(7):e0255068. doi: 10.1371/journal.pone.0255068 (PMC8321371; doi:10.1371/journal.pone.0255068)
Supplement: S3 File — (PDF) [file pone.0255068.s003.pdf]

S3 File. Behavioral results of EmpaToM in Study 2

Table 1

Group		CG				EG			
Emotionality		neutral		negative		neutral		negative	
ToM Requirement		non ToM	ToM	non ToM	ToM	non ToM	ToM	non ToM	ToM
Affect Rating	m	0.63	0.47	-1.43	-1.43	0.48	0.40	-1.65	-1.64
	sd	0.59	0.59	1.08	1.08	0.36	0.48	0.62	0.63
Compassion Rating	m	2.09	2.31	4.45	4.47	2.09	2.48	4.45	4.52
	sd	1.10	0.95	0.69	0.80	1.14	0.94	0.85	0.80
Accuracy	m	0.68	0.63	0.66	0.73	0.70	0.72	0.60	0.76
	sd	0.19	0.17	0.18	0.13	0.14	0.12	0.19	0.11
Confidence Rating	m	4.13	3.82	4.01	4.05	4.19	4.05	4.05	4.43
	sd	0.84	0.85	0.76	0.83	0.78	0.71	0.90	0.76
