# Supplementary material for: The influence of anger on empathy and theory of mind
Source: PLoS One. 2021 Jul 29;16(7):e0255068. doi: 10.1371/journal.pone.0255068 (PMC8321371; doi:10.1371/journal.pone.0255068)
Supplement: S4 File — (PDF) [file pone.0255068.s004.pdf]

S4 File. Task Effects in EmpaToM Study 2: The Reviewer

Table 1

ANOVA results for EmpaToM variable "Affect Rating"

Predictor	df_{Num}	df_{Den}	SS_{Num}	SS_{Den}	F	p	η^2_g
(Intercept)	1	47	54.95	53.39	48.37	.000	.37
Group	1	47	1.28	53.39	1.12	.294	.01
Emotionality	1	47	203.08	35.05	272.30	.000	.69
ToMRequirement	1	47	0.17	2.15	3.74	.059	.00
Group x Emotionality	1	47	0.15	35.05	0.20	.660	.00
Group x ToMRequirement	1	47	0.02	2.15	0.46	.503	.00
Emotionality x ToMRequirement	1	47	0.17	2.13	3.75	.059	.00
Group x Emotionality x ToMRequirement	1	47	0.02	2.13	0.34	.565	.00

Note. df_{Num} indicates degrees of freedom numerator. df_{Den} indicates degrees of freedom denominator. SS_{Num} indicates sum of squares numerator. SS_{Den} indicates sum of squares denominator. η^2_g indicates generalized eta-squared.

Table 2

ANOVA results for EmpaToM variable "Compassion Rating"

Predictor	df_{Num}	df_{Den}	SS_{Num}	SS_{Den}	F	p	η^2_g
(Intercept)	1	47	2215.68	105.07	991.08	.000	.93
Group	1	47	0.16	105.07	0.07	.791	.00
Emotionality	1	47	243.42	46.26	247.29	.000	.60
ToMRequirement	1	47	1.59	3.81	19.66	.000	.01
Group x Emotionality	1	47	0.05	46.26	0.05	.825	.00
Group x ToMRequirement	1	47	0.15	3.81	1.81	.185	.00
Emotionality x ToMRequirement	1	47	0.84	4.51	8.79	.005	.01
Group x Emotionality x ToMRequirement	1	47	0.05	4.51	0.51	.477	.00

Note. df_{Num} indicates degrees of freedom numerator. df_{Den} indicates degrees of freedom denominator. SS_{Num} indicates sum of squares numerator. SS_{Den} indicates sum of squares denominator. η^2_g indicates generalized eta-squared.

Table 3

ANOVA results for EmpaToM variable "Accuracy"

Predictor	df_{Num}	df_{Den}	SS_{Num}	SS_{Den}	F	p	η^2_g
(Intercept)	1	47	92.07	1.91	2268.0 7	.000	.95
Group	1	47	0.02	1.91	0.41	.526	.00
Emotionality	1	47	0.00	0.59	0.00	1..0	.00
ToMRequirement	1	47	0.14	1.20	5.35	.025	.03
Group x Emotionality	1	47	0.05	0.59	4.14	.048	.01
Group x ToMRequirement	1	47	0.06	1.20	2.44	.125	.01
Emotionality x ToMRequirement	1	47	0.22	0.74	13.65	.001	.05
Group x Emotionality x ToMRequirement	1	47	0.00	0.74	0.04	.848	.00

Note. df_{Num} indicates degrees of freedom numerator. df_{Den} indicates degrees of freedom denominator. SS_{Num} indicates sum of squares numerator. SS_{Den} indicates sum of squares denominator. η^2_g indicates generalized eta-squared.

Table 4

ANOVA results for EmpaToM variable "Confidence Rating"

Predictor	df_{Num}	df_{Den}	SS_{Num}	SS_{Den}	F	p	η^2_g
(Intercept)	1	47	3295.4 2	81.24	1906.5 8	.000	.96
Group	1	47	1.49	81.24	0.86	.357	.01
Emotionality	1	47	0.42	11.77	1.67	.202	.00
ToMRequirement	1	47	0.00	17.34	0.01	.932	.00
Group x Emotionality	1	47	0.05	11.77	0.19	.664	.00
Group x ToMRequirement	1	47	0.81	17.34	2.21	.144	.01
Emotionality x ToMRequirement	1	47	2.47	11.14	10.40	.002	.02
Group x Emotionality x ToMRequirement	1	47	0.09	11.14	0.38	.538	.00

Note. df_{Num} indicates degrees of freedom numerator. df_{Den} indicates degrees of freedom denominator. SS_{Num} indicates sum of squares numerator. SS_{Den} indicates sum of squares denominator. η^2_g indicates generalized eta-squared.

Table 5

2x2x2x12 ANOVA results for EmpaToM variable "Affect Rating" with added factor "Time"

Predictor	df_{Num}	df_{Den}	*Epsilon*	SS_{Num}	SS_{Den}	*F*	*p*	η^2_g
(Intercept)	1.00	47.00		659.46	640.73	48.37	.000	.21
Group	1.00	47.00		15.33	640.73	1.12	.294	.01
Emotionality	1.00	47.00		2436.90	420.63	272.30	.000	.50
ToMRequirement	1.00	47.00		2.05	25.75	3.74	.059	.00
Group x Emotionality	1.00	47.00		1.76	420.63	0.20	.660	.00
Group x ToMRequirement	1.00	47.00		0.25	25.75	0.46	.503	.00
Emotionality x ToMRequirement	1.00	47.00		2.04	25.55	3.75	.059	.00
Group x Emotionality x ToMRequirement	1.00	47.00		0.18	25.55	0.34	.565	.00
Time	8.31	390.74	0.76	2.77	346.59	0.38	.938	.00
Group x Time	8.31	390.74	0.76	1.97	346.59	0.27	.979	.00
Emotionality x Time	7.50	352.39	0.68	2.74	342.15	0.38	.925	.00
ToMRequirement x Time	8.21	385.88	0.75	9.95	326.72	1.43	.179	.00
Group x Emotionality x Time	7.50	352.39	0.68	6.27	342.15	0.86	.544	.00
Group x ToMRequirement x Time	8.21	385.88	0.75	11.29	326.72	1.62	.114	.00
Emotionality x ToMRequirement x Time	8.09	380.40	0.74	5.91	307.18	0.90	.514	.00
Group x Emotionality x ToMRequirement x Time	8.09	380.40	0.74	6.11	307.18	0.93	.488	.00

Note. df_{Num} indicates degrees of freedom numerator. df_{Den} indicates degrees of freedom denominator. Epsilon indicates Greenhouse-Geisser multiplier for degrees of freedom, *p*-values and degrees of freedom in the table incorporate this correction. SS_{Num} indicates sum of squares numerator. SS_{Den} indicates sum of squares denominator. η^2_g indicates generalized eta-squared.

Table 6

2x2x2x12 ANOVA results for EmpaToM variable "Compassion Rating" with added factor "Time"

Predictor	df_{Num}	df_{Den}	*Epsilon*	SS_{Num}	SS_{Den}	*F*	*p*	η^2_g
(Intercept)	1.00	47.00		26588.07	1260.88	991.08	.000	.86
Group	1.00	47.00		1.91	1260.88	0.07	.791	.00
Emotionality	1.00	47.00		2920.99	555.15	247.29	.000	.40
ToMRequirement	1.00	47.00		19.12	45.72	19.66	.000	.00
Group x Emotionality	1.00	47.00		0.59	555.15	0.05	.825	.00
Group x ToMRequirement	1.00	47.00		1.76	45.72	1.81	.185	.00
Emotionality x ToMRequirement	1.00	47.00		10.12	54.10	8.79	.005	.00
Group x Emotionality x ToMRequirement	1.00	47.00		0.59	54.10	0.51	.477	.00
Time	7.92	372.22	0.72	6.38	657.44	0.46	.885	.00
Group x Time	7.92	372.22	0.72	26.86	657.44	1.92	.057	.01
Emotionality x Time	8.47	397.89	0.77	13.23	647.90	0.96	.470	.00
ToMRequirement x Time	8.27	388.73	0.75	16.30	537.66	1.43	.182	.00
Group x Emotionality x Time	8.47	397.89	0.77	11.08	647.90	0.80	.606	.00
Group x ToMRequirement x Time	8.27	388.73	0.75	11.68	537.66	1.02	.420	.00
Emotionality x ToMRequirement x Time	8.06	378.86	0.73	10.12	620.65	0.77	.633	.00
Group x Emotionality x ToMRequirement x Time	8.06	378.86	0.73	19.78	620.65	1.50	.156	.00

Note. df_{Num} indicates degrees of freedom numerator. df_{Den} indicates degrees of freedom denominator. Epsilon indicates Greenhouse-Geisser multiplier for degrees of freedom, *p*-values and degrees of freedom in the table incorporate this correction. SS_{Num} indicates sum of squares numerator. SS_{Den} indicates sum of squares denominator. η^2_g indicates generalized eta-squared.

Table 7

2x2x2x12 ANOVA results for EmpaToM variable "Accuracy" with added factor "Time"

Predictor	df_{Num}	df_{Den}	*Epsilon*	SS_{Num}	SS_{Den}	*F*	*p*	η^2_g
(Intercept)	1.00	47.00		1104.82	22.89	2268.06	.000	.70
Group	1.00	47.00		0.20	22.89	0.41	.526	.00
Emotionality	1.00	47.00		0.00	7.05	0.00	1..0	.00
ToMRequirement	1.00	47.00		1.63	14.37	5.35	.025	.00
Group x Emotionality	1.00	47.00		0.62	7.05	4.14	.048	.00
Group x ToMRequirement	1.00	47.00		0.75	14.37	2.44	.125	.00
Emotionality x ToMRequirement	1.00	47.00		2.59	8.91	13.65	.001	.01
Group x Emotionality x ToMRequirement	1.00	47.00		0.01	8.91	0.04	.848	.00
Time	8.99	422.61	0.82	3.48	102.18	1.60	.112	.01
Group x Time	8.99	422.61	0.82	6.92	102.18	3.18	.001	.01
Emotionality x Time	9.02	423.82	0.82	3.06	111.85	1.29	.242	.01
ToMRequirement x Time	9.14	429.49	0.83	2.65	108.02	1.15	.323	.01
Group x Emotionality x Time	9.02	423.82	0.82	2.92	111.85	1.23	.276	.01
Group x ToMRequirement x Time	9.14	429.49	0.83	1.08	108.02	0.47	.898	.00
Emotionality x ToMRequirement x Time	8.88	417.30	0.81	0.94	103.26	0.43	.917	.00
Group x Emotionality x ToMRequirement x Time	8.88	417.30	0.81	1.79	103.26	0.82	.600	.00

Note. df_{Num} indicates degrees of freedom numerator. df_{Den} indicates degrees of freedom denominator. Epsilon indicates Greenhouse-Geisser multiplier for degrees of freedom, *p*-values and degrees of freedom in the table incorporate this correction. SS_{Num} indicates sum of squares numerator. SS_{Den} indicates sum of squares denominator. η^2_g indicates generalized eta-squared.

Table 8

2x2x2x12 ANOVA results for EmpaToM variable "Compassion Rating" with added factor "Time"

Predictor	df_{Num}	df_{Den}	*Epsilon*	SS_{Num}	SS_{Den}	*F*	*p*	η^2_g
(Intercept)	1.00	47.00		26588.07	1260.88	991.08	.000	.86
Group	1.00	47.00		1.91	1260.88	0.07	.791	.00
Emotionality	1.00	47.00		2920.99	555.15	247.29	.000	.40
ToMRequirement	1.00	47.00		19.12	45.72	19.66	.000	.00
Group x Emotionality	1.00	47.00		0.59	555.15	0.05	.825	.00
Group x ToMRequirement	1.00	47.00		1.76	45.72	1.81	.185	.00
Emotionality x ToMRequirement	1.00	47.00		10.12	54.10	8.79	.005	.00
Group x Emotionality x ToMRequirement	1.00	47.00		0.59	54.10	0.51	.477	.00
Time	7.92	372.22	0.72	6.38	657.44	0.46	.885	.00
Group x Time	7.92	372.22	0.72	26.86	657.44	1.92	.057	.01
Emotionality x Time	8.47	397.89	0.77	13.23	647.90	0.96	.470	.00
ToMRequirement x Time	8.27	388.73	0.75	16.30	537.66	1.43	.182	.00
Group x Emotionality x Time	8.47	397.89	0.77	11.08	647.90	0.80	.606	.00
Group x ToMRequirement x Time	8.27	388.73	0.75	11.68	537.66	1.02	.420	.00
Emotionality x ToMRequirement x Time	8.06	378.86	0.73	10.12	620.65	0.77	.633	.00
Group x Emotionality x ToMRequirement x Time	8.06	378.86	0.73	19.78	620.65	1.50	.156	.00

Note. df_{Num} indicates degrees of freedom numerator. df_{Den} indicates degrees of freedom denominator. Epsilon indicates Greenhouse-Geisser multiplier for degrees of freedom, *p*-values and degrees of freedom in the table incorporate this correction. SS_{Num} indicates sum of squares numerator. SS_{Den} indicates sum of squares denominator. η^2_g indicates generalized eta-squared.
