# Supplementary material for: The influence of anger on empathy and theory of mind
Source: PLoS One. 2021 Jul 29;16(7):e0255068. doi: 10.1371/journal.pone.0255068 (PMC8321371; doi:10.1371/journal.pone.0255068)
Supplement: S5 File — (PDF) [file pone.0255068.s005.pdf]

S5 File. Behavioral results of EmpaToM in

Study 3 Table 1

Group		CG				EG			
Emotionality		neutral		negative		neutral		negative	
ToM Requirement		non ToM	ToM	non ToM	ToM	non ToM	ToM	non ToM	ToM
Affect Rating	m	0.55	0.59	-1.35	-1.40	0.47	0.49	-1.36	-1.42
	sd	0.38	0.39	0.70	0.69	0.55	0.36	0.65	0.62
Compassion Rating	m	2.24	2.54	4.51	4.52	1.83	2.04	4.47	4.46
	sd	0.97	0.95	0.64	0.60	1.24	1.06	0.59	0.55
Accuracy	m	0.77	0.75	0.68	0.83	0.75	0.71	0.66	0.74
	sd	0.14	0.10	0.12	0.13	0.14	0.17	0.19	0.15
Confidence Rating	m	4.37	3.94	3.85	4.07	4.26	4.14	4.13	4.27
	sd	0.66	0.48	0.55	0.77	0.69	0.53	0.63	0.59
