# Supplementary material for: The influence of anger on empathy and theory of mind
Source: PLoS One. 2021 Jul 29;16(7):e0255068. doi: 10.1371/journal.pone.0255068 (PMC8321371; doi:10.1371/journal.pone.0255068)
Supplement: S7 File — (PDF) [file pone.0255068.s007.pdf]

S7 File. Task Effects in EmpaToM: Pooled data of Studys 2 and 3

Table 1

ANOVA results for EmpaToM variable "Affect Rating"

Predictor	df_{Num}	df_{Den}	SS_{Num}	SS_{Den}	F	p	η^2_g
(Intercept)	1	91	88.30	70.72	113.63	.000	.37
Group	1	91	1.13	70.72	1.45	.231	.01
Study	1	91	0.90	70.72	1.15	.286	.01
Emotionality	1	91	369.52	69.45	484.19	.000	.71
ToMRequirement	1	91	0.14	3.43	3.59	.061	.00
Group x Study	1	91	0.28	70.72	0.36	.551	.00
Group x Emotionality	1	91	0.01	69.45	0.01	.916	.00
Study x Emotionality	1	91	0.41	69.45	0.53	.467	.00
Group x ToMRequirement	1	91	0.00	3.43	0.13	.719	.00
Study x ToMRequirement	1	91	0.05	3.43	1.23	.271	.00
Emotionality x ToMRequirement	1	91	0.01	3.89	0.25	.620	.00
Group x Study x Emotionality	1	91	0.21	69.45	0.27	.605	.00
Group x Study x ToMRequirement	1	91	0.02	3.43	0.48	.489	.00
Group x Emotionality x ToMRequirement	1	91	0.01	3.89	0.19	.665	.00
Study x Emotionality x ToMRequirement	1	91	0.24	3.89	5.55	.021	.00
Group x Study x Emotionality x ToMRequirement	1	91	0.01	3.89	0.17	.683	.00

Note. df_{Num} indicates degrees of freedom numerator. df_{Den} indicates degrees of freedom denominator. SS_{Num} indicates sum of squares numerator. SS_{Den} indicates sum of squares denominator. η^2_g indicates generalized eta-squared.

Table 2

ANOVA results for EmpaToM variable "Compassion Rating"

Predictor	df_{Num}	df_{Den}	SS_{Num}	SS_{Den}	F	p	η^2_g
(Intercept)	1	91	4238.24	175.18	2201.67	.000	.94
Group	1	91	0.83	175.18	0.43	.514	.00
Study	1	91	0.21	175.18	0.11	.741	.00
Emotionality	1	91	496.06	98.65	457.61	.000	.63
ToMRequirement	1	91	2.24	7.90	25.75	.000	.01
Group x Study	1	91	2.28	175.18	1.19	.279	.01
Group x Emotionality	1	91	0.64	98.65	0.59	.444	.00
Study x Emotionality	1	91	0.33	98.65	0.30	.584	.00
Group x ToMRequirement	1	91	0.03	7.90	0.31	.579	.00
Study x ToMRequirement	1	91	0.07	7.90	0.84	.362	.00
Emotionality x ToMRequirement	1	91	1.61	9.24	15.86	.000	.01
Group x Study x Emotionality	1	91	1.31	98.65	1.20	.275	.00
Group x Study x ToMRequirement	1	91	0.15	7.90	1.68	.199	.00
Group x Emotionality x ToMRequirement	1	91	0.01	9.24	0.06	.811	.00
Study x Emotionality x ToMRequirement	1	91	0.00	9.24	0.00	.978	.00
Group x Study x Emotionality x ToMRequirement	1	91	0.06	9.24	0.57	.453	.00

Note. df_{Num} indicates degrees of freedom numerator. df_{Den} indicates degrees of freedom denominator. SS_{Num} indicates sum of squares numerator. SS_{Den} indicates sum of squares denominator. η^2_g indicates generalized eta-squared.

Table 3

ANOVA results for EmpaToM variable "Accuracy"

Predictor	df_{Num}	df_{Den}	SS_{Num}	SS_{Den}	F	p	η^2_g
(Intercept)	1	91	191.25	3.85	4516.82	.000	.96
Group	1	91	0.01	3.85	0.19	.668	.00
Study	1	91	0.23	3.85	5.52	.021	.03
Emotionality	1	91	0.01	1.11	0.54	.465	.00
ToMRequirement	1	91	0.21	1.88	10.14	.002	.02
Group x Study	1	91	0.08	3.85	1.80	.183	.01
Group x Emotionality	1	91	0.05	1.11	3.97	.049	.01
Study x Emotionality	1	91	0.01	1.11	0.60	.442	.00
Group x ToMRequirement	1	91	0.01	1.88	0.31	.576	.00
Study x ToMRequirement	1	91	0.00	1.88	0.16	.689	.00
Emotionality x ToMRequirement	1	91	0.43	1.38	28.27	.000	.05
Group x Study x Emotionality	1	91	0.01	1.11	0.81	.372	.00
Group x Study x ToMRequirement	1	91	0.08	1.88	3.67	.059	.01
Group x Emotionality x ToMRequirement	1	91	0.00	1.38	0.07	.795	.00
Study x Emotionality x ToMRequirement	1	91	0.00	1.38	0.00	.951	.00
Group x Study x Emotionality x ToMRequirement	1	91	0.00	1.38	0.30	.583	.00

Note. df_{Num} indicates degrees of freedom numerator. df_{Den} indicates degrees of freedom denominator. SS_{Num} indicates sum of squares numerator. SS_{Den} indicates sum of squares denominator. η^2_g indicates generalized eta-squared.

Table 4

ANOVA results for EmpaToM variable "Confidence"

Predictor	df_{Num}	df_{Den}	SS_{Num}	SS_{Den}	F	p	η^2_g
(Intercept)	1	91	6441.39	124.37	4712.97	.000	.97
Study	1	91	0.12	124.37	0.09	.766	.00
Group	1	91	2.39	124.37	1.75	.189	.01
Emotionality	1	91	0.00	17.56	0.01	.927	.00
ToMRequirement	1	91	0.02	27.86	0.07	.789	.00
Study x Group	1	91	0.03	124.37	0.02	.891	.00
Study x Emotionality	1	91	0.78	17.56	4.04	.047	.00
Group x Emotionality	1	91	0.40	17.56	2.09	.152	.00
Study x ToMRequirement	1	91	0.05	27.86	0.16	.690	.00
Group x ToMRequirement	1	91	0.87	27.86	2.83	.096	.00
Emotionality x ToMRequirement	1	91	4.65	18.99	22.30	.000	.02
Study x Group x Emotionality	1	91	0.12	17.56	0.60	.441	.00
Study x Group x ToMRequirement	1	91	0.11	27.86	0.37	.545	.00
Study x Emotionality x ToMRequirement	1	91	0.00	18.99	0.01	.943	.00
Group x Emotionality x ToMRequirement	1	91	0.06	18.99	0.29	.589	.00
Study x Group x Emotionality x ToMRequirement	1	91	0.47	18.99	2.27	.135	.00

Note. df_{Num} indicates degrees of freedom numerator. df_{Den} indicates degrees of freedom denominator. SS_{Num} indicates sum of squares numerator. SS_{Den} indicates sum of squares denominator. η^2_g indicates generalized eta-squared.
